# Numerical Simulation of the Effect of Freeze-Thaw Cycles on the Axial Compression Strength of Rubber Concrete

**DOI:** 10.3390/ma16124460

**Published:** 2023-06-19

**Authors:** Dingyi Hao, Xiaoyu Huang, Houmin Li, Zhou Cao, Zijiang Yang, Xianfeng Pei, Kai Min, Cai Liu, Wenchao Li, En Zhang, Jie Shen

**Affiliations:** 1 School of Engineering, Architecture and The Environment, Hubei University of Technology, Wuhan 430068, China; 2 China Construction Third Bureau First Engineering Co., Ltd., Wuhan 430040, Chinarosrs520@163.com (E.Z.); 3 Wuhan Construction Engineering Company Limited, Wuhan 430056, China

**Keywords:** rubber concrete, uniaxial compression, FTCs, numerical simulation

## Abstract

The incorporation of rubber can enhance concrete’s durability and effectively reduce the damage caused by freeze-thaw cycling (FTC). Still, there has been only limited research on the damage mechanism of RC at the fine view level. To gain insight into the expansion process of uniaxial compression damage cracks in rubber concrete (RC) and summarize the internal temperature field distribution law during FTC, a fine RC thermodynamic model containing mortar, aggregate, rubber, water, and interfacial transition zone (ITZ) is established in this paper, and the cohesive element is selected for the ITZ part. The model can be used to study the mechanical properties of concrete before and after FTC. The validity of the calculation method was verified by comparing the calculated results of the compressive strength of concrete before and after FTC with the experimental results. On this basis, this study analyzed the compressive crack extension and internal temperature distribution of RC at 0, 5, 10, and 15% replacement rates before and after 0, 50, 100, and 150 cycles of FTC. The results showed that the fine-scale numerical simulation method can effectively reflect the mechanical properties of RC before and after FTC, and the computational results verify the applicability of the method to rubber concrete. The model can effectively reflect the uniaxial compression cracking pattern of RC before and after FTC. Incorporating rubber can impede temperature transfer and reduce the compressive strength loss caused by FTC in concrete. The FTC damage to RC can be reduced to a greater extent when the rubber incorporation is 10%.

## 1. Introduction

With accelerating urbanization, the center of gravity of China’s development has shifted from the original coastal cities to the alpine regions in the northwest. Influenced by environmental factors, such as high altitudes and large temperature differences between day and night, many infrastructures in these areas are subjected to freeze-thaw damage [1]. Freeze-thaw damage is a thermodynamically coupled change process consisting of several factors [2], and it includes three main stages: water filling, water freezing, and expansion cracking [3]. External water enters the concrete interior to fill the pores, allowing water to accumulate inside the concrete. When the external temperature decreases until it reaches freezing, the internal water changes into ice with a 9% expansion rate, thus causing tensile stress in the concrete internal pore wall. If the stress value is greater than the tensile strength, the concrete will produce micro-cracks, and as the temperature increases, the internal ice melts, and the tensile stress is naturally released. It is not difficult to find that such a continuous cycle will cause gradually larger concrete cracks and thus produce deterioration. Under the combined action of chloride ions, carbonate ions, and external loads [4], buildings are highly susceptible to deterioration and durability damage, seriously threatening the safety of people’s lives and property. In 1945, Powers proposed the hydrostatic pressure theory [5,6], which provided new ideas for scholars to further study how to reduce the phenomenon of freeze-thaw damage, and a large number of scholars thus started research on the mechanism of freeze-thaw cycle (FTC) damage [7,8,9] to better reduce the negative effects of FTC damage to buildings.

In the 1990s, Eldin and Senouci conducted preliminary research on rubber concrete (RC) as a new material. They found that adding rubber to concrete can, on the one hand, reduce the pressure caused by the environmental damage caused by discarded tires; on the other hand, adding rubber improves concrete’s durability to a certain extent [10]. Based on this finding, domestic and foreign scholars conducted multiple experiments [11,12,13,14] and concluded that, although the addition of rubber weakens the compressive and splitting mechanical properties of concrete to a certain extent, the durability of concrete has been significantly improved. RC has good impermeability, frost resistance, and chloride ion resistance, coupled with the low water absorption of rubber itself, making it more suitable for use in high-altitude and cold regions. Some scholars have also focused their research on the size of rubber particles and the mechanism of freeze-thaw resistance. For example, Xu et al. attempted to use rubber particles instead of fine aggregates [15] to discuss the mechanical properties of RC after FTCs. They pointed out that adding rubber to concrete can effectively reduce FTC damage, the rubber particle size is inversely proportional to the concrete’s ability to resist internal damage, and it is directly proportional to the concrete quality damage rate. Gou et al. used vibrating wire strain sensors to monitor the strain of reinforced concrete during the freeze-thaw process in real time [16]. On the one hand, they summarized the relationship between the FTC temperature of rubber powder particle concrete and macroscopic changes, such as relative dynamic elastic modulus and mass loss. On the other hand, they found that rubber can effectively prevent external water from entering the interior of concrete, thereby reducing the internal expansion force caused by water condensation into ice and further preventing the generation of concrete cracks caused by temperature stress. Richardson et al. conducted comparative experiments on five different sizes of rubber particles based on the relevant characteristics of concrete, such as air content and freeze-thaw durability. The conclusion drawn from the analysis was that there is no significant correlation between the size of rubber particles and the compressive strength of concrete. Rubber shavings with a diameter smaller than 0.5 mm provide better protection for concrete during FTCs [17]. In addition, relevant scholars have conducted the latest research using rubber materials in engineering. Al-Fakih used rubber debris and fly ash to replace some fine aggregates [18], producing rubber concrete-interlocking masonry with good ductility. Finite element analysis was used to analyze the deformation of hollow or grouted masonry, which is beneficial for practical engineering applications. Crumb rubber can also effectively improve the ductility, Poisson’s ratio, and fluidity of engineering cement-based composites (ECCs), and adding inorganic materials, such as nanosilica and graphite oxide can effectively alleviate the poor mechanical properties caused by crumb rubber and produce a large number of microholes when combined with cementitious materials [19]. Shaaban et al. treated its surface with sodium hydroxide before using crumb rubber [20]. After studying mixed concrete composed of steel fibers and crumb rubber, he found that adding a small number of steel fibers can effectively improve the degradation effect of rubber on the compressive strength and other mechanical properties of concrete. Rubber concrete will also be more widely used in practical engineering through continuous improvement and development.

Numerical simulations can gradually replace complex FTC experiments due to the rapid development of computer simulation technology and the in-depth study of the coupling theory of fracture mechanics and thermodynamics. This phenomenon has greatly reduced the experimental time and has alleviated the environmental damage caused by the extraction of raw materials. Numerical simulation of FTCs is the process of simulating computer models of concrete using the relevant theories of fracture mechanics and size effects. By calculating the temperature distribution inside concrete and the crack development after mechanical experiments, it is possible to derive how the variable factors affect the mechanical properties of concrete after FTCs. To improve the realism of numerical simulations, related scholars have established multi-scale computational models using the relevant principles of pore mechanics and thermodynamics and have compiled the laws of the damage evolution process of FTCs. Duan [21] established a set of control models for the FTC process and fitted the differential equations of concrete freeze-thaw damage by calculations to derive the distribution of temperature fields, deformation, and the magnitude of pore pressure caused by water freezing in concrete under FTCs. Yu [22] developed a two-dimensional three-phase computational model using Python in ABAQUS to simulate uniaxial stresses on concrete before and after FTCs. She concluded that FTC damage affects the mechanical properties of concrete, while finding that coarse aggregates and interfacial transition zones (ITZs) will play a role in the FTC process. Li et al. used a fiber model to simulate the effects of FTCs on the durability of concrete under salt-frost erosion [23], using the relative dynamic modulus of elasticity as the reference standard. By simulating the temperature field obtained, we can identify and reduce the internal temperature changes caused by FTCs and their adverse effects, providing the relevant theoretical basis for subsequent research on concrete durability. Bharadwaj et al. [24], to verify the extent of the influence of pores inside concrete during FTCs, developed the pore distribution model (PPMC) by analyzing the Power model, OPC kinetic model, and related parameters, and the estimation of the secondary adsorption rate were used to make the model more effective in predicting critical saturation, leading to the conclusion that the pore volume was positively correlated with the freezing resistance of concrete within a reasonable range.

However, many scholars have studied the numerical simulation of FTCs without considering the effect of water or ice effect, and the numerical simulation of FTCs of RC is still in the early stage. In this paper, we analyzed the FTC models of many scholars, established a two-dimensional mesoscopic calculation model of multiphase concrete, and added a cohesive force element as the interface transition zone to investigate the effect of rubber admixture on the FTC of concrete. The analytical results were compared with relevant experimental data to determine the relationship between the rubber content and the rate of compressive strength loss after FTCs, verifying the numerical simulation’s feasibility. The model also provides a relevant theoretical study for the use of RC in alpine regions.

## 2. Model Establishment

### 2.1. Aggregate Building

This study used a stochastic aggregate model for numerical simulation calculations. In the mesoscale model, RC is usually considered a multiphase material consisting of mortar matrix, coarse aggregate, mortar-coarse aggregate ITZ, rubber particles, mortar-rubber ITZ, and water, and each aggregate is considered an isotropic material in the model design. Aggregate diameter was chosen from 5–10 mm, 10–15 mm, and 15–20 mm, following the random aggregate placement principle and using Fuller’s gradation to achieve the best performance of the generated RC model [25].

(1) $$P=100\sqrt{\frac{D_{0}}{D_{m}}}$$

P—Percentage of aggregate passing through sieve diameter $D_{0}$ to total aggregate;

$D_{0}$—Diameter of sieve holes;

$D_{m}$—The maximum diameter of the aggregate. The maximum diameter of the aggregate selected for this model was 20 mm, consistent with the selection of comparison experiments.

The Ravavin formulation is a three-dimensional concrete computational model transformed into a two-dimensional planar model for relevant research calculations [26], ensuring the reasonableness of the grading inside the model to obtain the probability of any aggregate diameter D within the two-dimensional concrete.

(2) $$P\left(D<D_{0}\right)=P_{K}[1.065{\left(\frac{D_{0}}{D_{m}}\right)}^{0.5}-0.053{\left(\frac{D_{0}}{D_{m}}\right)}^{4}-0.012{\left(\frac{D_{0}}{D_{m}}\right)}^{6}-0.0045{\left(\frac{D_{0}}{D_{m}}\right)}^{8}+0.0025{\left(\frac{D_{0}}{D_{m}}\right)}^{10}]$$

$P_{K}—$The overall percentage of aggregate volume. The $P_{K}$ selected for this paper is 0.6.

**Figure 1:**
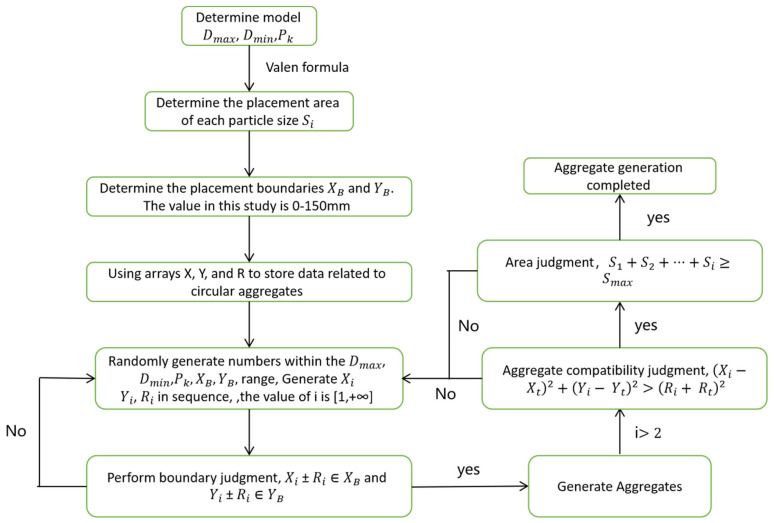
Flow chart of model aggregate generation.

**Figure 2:**
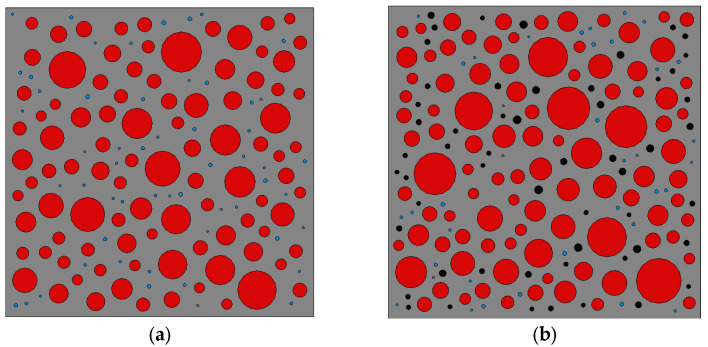

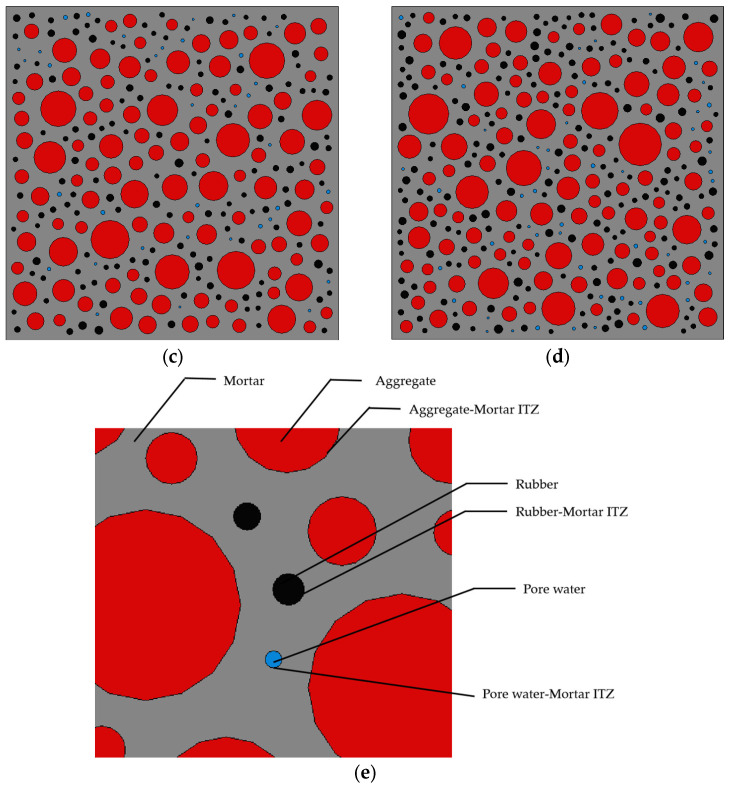
Aggregate generation diagram with different rubber substitution rates: (**a**) 0% rubber substitution rate; (**b**) 5% rubber substitution rate; (**c**) 10% rubber substitution rate; (**d**) 15% rubber substitution rate; (**e**) model partial enlargement.

Aggregate generation code was written in the Python language. Before the aggregates are generated, it is necessary to determine the model boundary range first, the maximum and minimum particle sizes of the generated aggregates, and rubber, $P_{K}$, to divide the aggregates in that range into intervals. Once these factors have been determined, the area occupied by each interval of aggregate and rubber is calculated in a Microsoft Excel table using Equation (2) and filled into Python. ABAQUS2021 contains a Python language adapter that, when read, first generates the model boundary; the second step randomly generates the x- and y-axis circular coordinates of the circular aggregate and the radius r, which are stored in the defined group X, Y, and R. The third step is to randomly assign values from the first. The third step randomly assigns values from the first interval range to the array to obtain the relevant data of the first aggregate, and then it judges the boundary of these data, which cannot exceed the model boundary range, indicating that the first aggregate is generated. Based on the above method of generating subsequent aggregates, the overlap between aggregates is judged, and the distance between aggregates and the center of the aggregate circle should be greater than the sum of the radius of the two aggregates and the distance of the given range; any failure of the above judgment will delete the aggregate and regenerate it. When this interval generation meets the area requirement, it proceeds to the next interval for aggregate assignment. The generation ends when the generated aggregate area reaches the total aggregate area. The above steps are shown in Figure 1, and the rubber concrete generated by the above steps for each substitution rate is shown in Figure 2.

The flow chart of aggregate generation is as follows:

The use of Equation (2) to find the area of each aggregate is shown in Table 1:

Through the above steps, the distribution of the generated aggregates is calculated as follows:

### 2.2. Model Material Parameters

The two-dimensional random aggregate RC model is a composite model composed of multiple materials. The model mainly explores the effect of the rubber substitution rate on the FTC of RC and simulates the overall thermodynamic damage state of concrete using the thermodynamic properties of each phase material. The mortar matrix plays a crucial role in the mechanical properties of rubber concrete. Since the mortar matrix is considered concrete with a lower strength, it is regarded as a homogeneous and continuous elastic-plastic material during the modeling process, and a concrete plastic damage (CDP) model is used as the constitutive law of the mortar. It was shown [27] that considering rubber as a linear, hyperelastic, or viscoelastic material requires exploring rubber strain during uniaxial compression. Since the rubber strain inside the RC after being subjected to compression loading is small, the choice of linear elastic material can better match the properties of the rubber material. At sub-zero temperatures, the FTC is more obvious, ice is larger than water during heat transfer, and the freezing and swelling damage after the water is cold and frozen is the dominant damage. Considering the difficulty of quantifying the modulus of elasticity of water during uniaxial compression, the water parameters in this model are set in the same way as in [28], in which pore water was assumed to be pore ice and set in solid elements. In this study, to further restore the principle of ice operation, the ice elements were set to produce expansion at sub-zero temperatures, and the pore ice content was consistent with the pore water rate, which was set at 2%. Aggregate and rubber are consistently regarded as linear elastic parameters. At the same time, the ITZ part uses viscous elements to define the properties and render them close to the working and damage principles of ITZ. The overall model combines CDP and cohesion damage, a relatively new model damage setting. The relevant material parameters are shown in Table 2.

Aggregate shape can also have a large effect on the numerical simulation results. Finite element analysis and experimental data by Kim et al. [32] and Huang et al. [33] showed that polygonal aggregates can produce stress concentrations at sharp edges when they are subjected to tension or compression, causing the calculation results to deviate significantly. In addition, although circular aggregates may cause a slight increase in tensile and compressive strength, they do not affect the overall mechanical properties of the model to a large extent. To simplify the model calculation, the mechanical experiment of this study finally chooses the circular aggregate model for simulation and assumes that the rubber and pore water inside the concrete are circular models.

### 2.3. Model Comparison

In this study, the model was designed with reference to the thermodynamic models of Yu [22] and Peng et al. [34], both of whom used the compressive strength of concrete before and after elements as the basis for validation but with relatively novel changes in the selection and composition of the relevant element types. The two-dimensional mesoscale model used by Yu to analyze the change in compressive strength of ordinary concrete before and after elements did not consider the influence of pore water on the elements, and the interface transition zone used a more complex solid element. Peng used a three-dimensional model to analyze the elements of ordinary concrete, which simplified the freeze-thaw process and did not directly consider the influence of water. The model simplified the freeze-thaw process and did not directly consider the effect of water, and it also used solid cells to simulate the operation of the interface transition zone. The model in this study is based on the ordinary concrete model mixed with rubber particles, which has been rare in current research, and it generates pore water to participate in the cyclic temperature action, which is a more realistic reflection of internal temperature changes. More noteworthy is the use of cohesive elements as the interface transition zone; the overall model is a hybrid model combining solid and cohesive forces, expediting the calculation while more accurately simulating the working state of each part of the material.

### 2.4. Numerical Simulation Experiment Step Setting

**Figure 3:**
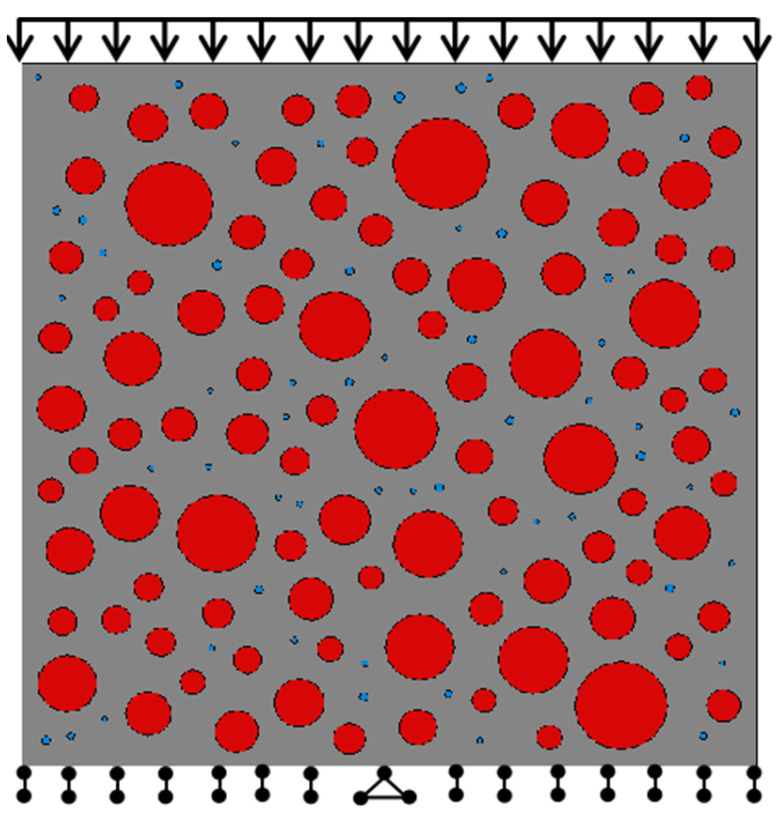
Loading sketch of uniaxial compression numerical simulation.

The numerical analysis of this model selected a 150 × 150 mm calculation model. The static uniaxial compression process was simulated in the Abaqus2021 standard analysis program. Geometric nonlinearity was chosen for the analysis step setting, with initial incremental steps of 0.01, a minimum value of 1 × 10^−15^, and a maximum value of 0.05. In this case, the model convergence is good. The direct vertical displacement load is applied to the upper nodes, which can avoid the load inertia factor affecting the compressive strength. The bottom restrains the movement in the y-axis direction, and the middle node is fixed by hinging, thereby restoring the pressurization of the real load-bearing machine. The loading process of single-axis compressive numerical simulation is shown in Figure 3.

### 2.5. Mesh Size Selection

**Figure 4:**
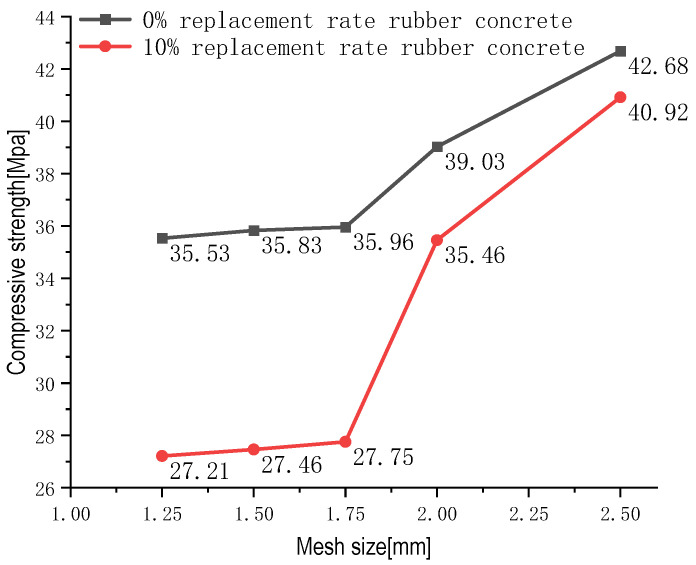
Compressive strength of RC with 0% and 10% substitution at different mesh sizes.

Many numerical simulation experiments have shown that the model’s mesh size and maximum particle size are relevant factors that must be considered during the calculation phase [35,36]. After remaining consistent with the control test situation, the model needs to focus on the influence of grid size on the calculation results. To avoid the adverse effects caused by the sensitivity of the model grid and to investigate the changes in the bearing capacity size before and after the FTC, the study therefore chose 2.5, 2, 1.75, 1.5, and 1.25 mm grids for the 0% rubber substitution rate and 10% rubber substitution rate models for the bearing capacity simulation analysis, and the calculated bearing capacity is shown in Figure 4. When the grid sizes are 2.5 and 2 mm, the generated grid effect is too large, and the deviation of the obtained bearing capacity is relatively serious. During the calculation process of multiple sets of models, some model strengths show discreteness, making it difficult to simulate more realistic experimental data. When the 1.75-mm mesh is used, the slope of the compressive strength curve does not change significantly, indicating that the bearing capacity gradually tends toward a stable state after the grid is reduced. The two models with rubber substitution rates of 0% and 10% showed little difference in the rate of change of compressive strength in the simulations with mesh sizes of 1.75 and 1.25 mm (1.2% and 1.9%, respectively), but the difference between them in the number of nodes was large (Table 3 and Table 4). To reduce the influence of grid sensitivity on the simulation results and improve the computer’s computational efficiency, the model finally chosen used a 1.75-mm mesh for the analysis.

### 2.6. ITZ Cohesive Model

Determining the ITZ in concrete computational models can accurately simulate micro-mechanical damage within the concrete, so it is especially important to accurately grasp the ITZ working mechanism and damage in mesoscale calculations. The current experiments on the ITZ mainly include macroscopic experiments to measure mechanical parameters or use micro-CT and nanoindentation [37]. Still, the measurement process is complex and costly, so numerical simulations have been used for related studies. In concrete, the most vulnerable part of the ITZ is the initial destruction. The ITZ is assumed to be a solid cell of mortar matrix with weak mechanical properties in the simulation stage. Therefore, most scholars choose 50–200 nm [38,39] as the thickness of the ITZ. Currently, the ITZ mainly uses solid elements or discrete elements. When using a solid element design, it is impossible to effectively restore the ITZ’s working principle in its actual state. However, although the design method of discrete elements can simulate the working state of the ITZ, the computational cost is still relatively high [40,41].

The model of this study uses cohesive elements as a zero-thickness ITZ, mainly including a mortar-coarse aggregate ITZ, a mortar-rubber ITZ, and a water-mortar contact zone. The thermal analysis is calculated using the viscous heat transfer element COH2D4T to analyze the temperature field distribution during each FTC and to restore the actual situation to a greater extent.

#### 2.6.1. Damage Initial Guidelines

**Figure 5:**
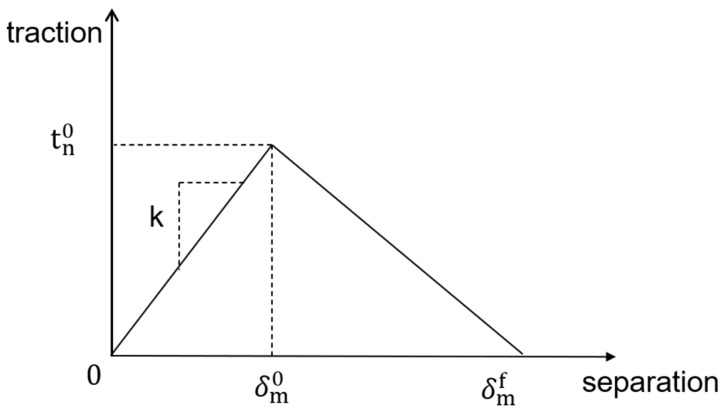
Cohesive traction separation of this composition.

The traction separation theory of cohesive elements is used to define failure and progressive damage in the cohesive layer (Figure 5), using a combination of damage mechanisms acting with the generated cohesive elements, using three parts of the damage initial criterion, the damage evolution form, and the removal of damaged elements to evaluate.

To accurately explore the failure and damage processes of various phase materials in the calculation process, the model introduces an initial damage criterion: the degradation of material point response. When a specific value or higher is reached, the damage starts. When the nominal stress ratio of the quadratic interaction function is 1, it is expressed as:

(3) $${\left\{\frac{\langle t_{n}\rangle }{t_{n}^{o}}\right\}}^{2}+{\left\{\frac{t_{s}}{t_{s}^{o}}\right\}}^{2}+{\left\{\frac{t_{t}}{t_{t}^{o}}\right\}}^{2}=1$$

where $\delta_{m}^{0}$ is the initial effective displacement of the damage, and $\delta_{m}^{f}$ is the full failure effective displacement.

A nominal strain ratio of 1 for the secondary interaction function is expressed as:

(4) $${\left\{\frac{\langle \varepsilon_{n}\rangle }{\varepsilon_{n}^{o}}\right\}}^{2}+{\left\{\frac{\varepsilon_{s}}{\varepsilon_{s}^{o}}\right\}}^{2}+{\left\{\frac{\varepsilon_{t}}{\varepsilon_{t}^{o}}\right\}}^{2}=1$$

where $t_{n}$, $t_{s}$, and $t_{t}$ are nominal traction stress vectors; $t_{n}^{o}$, $t_{s}^{o}$, and $t_{t}^{o}$ are nominal stress peaks perpendicular to the interface or in the first and second shear directions; $\varepsilon_{n}^{o}$, $\varepsilon_{s}^{o}$, and $\varepsilon_{t}^{o}$ are their nominal strain peaks; and the Macaulay symbol 〈〉 indicates that stress damage will not occur due to pure compression deformation.

#### 2.6.2. Damage Evolution Forms

The rapidity of the stiffness rate is described using damage evolution, which consists of two main parts. The first part consists of three factors: the initial effective displacement of damage $\delta_{m}^{0}$, the effective displacement of complete failure $\delta_{m}^{f}$, and the damage consumption energy $G^{C}$; while the second part is in the form of damage evolution of damage variables from the initial damage to the complete failure state in the mixed mode.

The fracture energy criterion of Benzeggagh–Kenane was used for the damage evolution.

(5) $$G_{n}^{C}+\left(G_{s}^{C}-G_{n}^{C}\right){\left\{\frac{G_{S}}{G_{T}}\right\}}^{\eta }=G_{C}$$

When $G_{n}^{C}$ is the first fracture energy, $G_{s}^{C}$ is the second fracture energy, $G_{S}=G_{s}+G_{t}$, and $G_{T}=G_{n}+G_{S}$, η is the mixed fracture energy coefficient.

Damage variable D is:

(6) $$D=\frac{\delta_{m}^{f}(\delta_{m}^{MAX}-\delta_{m}^{0})}{\delta_{m}^{MAX}(\delta_{m}^{f}-\delta_{m}^{0})}$$

where $\delta_{m}^{MAX}$ is the maximum effective displacement at loading, $\delta_{m}^{f}$ = $2G^{C}$/$T_{eff}^{0}$, and $T_{eff}^{0}$ is the initial effective traction force of the damage.

The relevant ITZ parameters are shown in Table 5.

### 2.7. Mortar Principal Structure Theory of the Model

**Figure 6:**
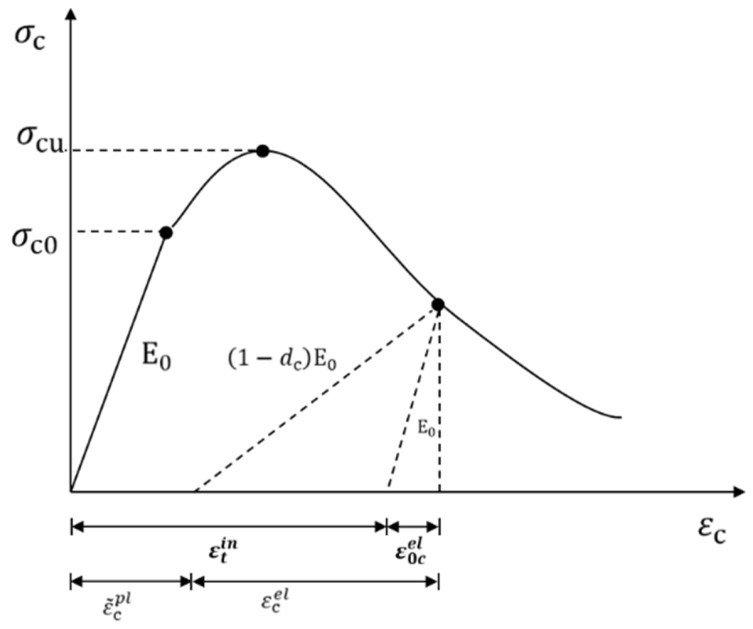
Uniaxial tensile stress–strain response.

**Figure 7:**
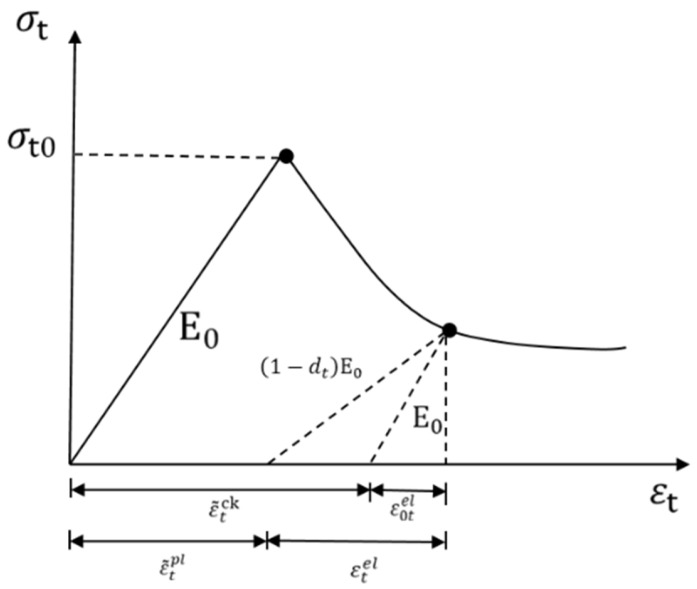
Uniaxial compression stress–strain response.

To better investigate the effect of FTC damage on the mortar part, the model treats the intrinsic relationship of the mortar part as a plastic damage intrinsic relationship and simulates the nonlinear response of the concrete material more realistically by calculating the damage accumulation in compression and tension of the model. The uniaxial tensile and compressive stress–strain responses are shown in Figure 6 and Figure 7.

The mortar stress–strain relationship is as follows:

(7) $$\sigma =\left(1-d\right)D_{0}^{el}\left(\varepsilon -\varepsilon^{pl}\right)$$

where d is the isotropic damage variable, $D_{0}^{el}$ is the initial linear elastic tensor, and $\varepsilon^{pl}$ is the plastic strain tensor.

The isotropic damage d variable can be divided into $d_{t}$ and $d_{c}$ with the expressions for the uniaxial tension-compression state:

(8) $$\sigma_{t}=\left(1-d_{t}\right)E_{0}\left(\varepsilon_{t}-\varepsilon_{t}^{pl}\right)$$

(9) $$\sigma_{c}=(1-d_{c})E_{0}(\varepsilon_{c}-\varepsilon_{c}^{pl})$$

where $E_{0}$ is the initial modulus of elasticity.$\varepsilon_{t}^{pl},\varepsilon_{c}^{pl}$ is the tensile and compressive plastic strain tensor. $d_{t}$, $d_{c}$ is subject to tensile, compressive damage variables, and both values are located between 0 and 1, with 1 indicating complete damage and 0 indicating no damage.

The focus of this study is to investigate the effect of FTC on RC in uniaxial compression using the change in compressive strength values before and after FTC. Lee et al. obtained the stress–strain conditions for concrete in uniaxial tension or compression based on the relevant equations to modify the intrinsic yield function [43]:

(10) $$F=\frac{1}{1-\alpha }(\bar{q}-3a\bar{q}+\beta (\varepsilon^{pl})\langle \bar{\sigma_{\max}}\rangle -r\langle \bar{\sigma_{\max}}\rangle -\overset{-}{\sigma_{c}}\varepsilon^{pl}$$

(11) $$\alpha =\frac{\left(\sigma_{b0}/\sigma_{c0}\right)-1}{2\left(\sigma_{b0}/\sigma_{c0}\right)-1}$$

(12) $$\beta =\frac{\bar{\sigma_{c0}}\left(\varepsilon_{c}^{pl}\right)}{\bar{\sigma_{t0}}\left(\varepsilon_{t}^{pl}\right)}\left(1-\alpha \right)-\left(1+\alpha \right)$$

(13) $$\gamma =\frac{3\left(1-K\right)}{2K-1}$$

where $\bar{\sigma_{max}}$ is the maximum effective stress, $\bar{q}$ is the equivalent force,$\sigma_{b0},\sigma_{c0}$ is the biaxial and uniaxial compressive strength, $\bar{\sigma_{c0}}$, $\bar{\sigma_{t0}}$ is the effective cohesive stress in compression and tension; and *K* is the ratio of the tensile and compressive radial first and stress invariants.

The equation of the stress–strain curve under pressure is:

(14) $$y=\frac{\sigma }{f_{c}}=\left\{\begin{array}{c} \alpha_{a}x+\left(3-2\alpha_{a}\right)x^{2}+\left(\alpha_{a}-2\right)x^{3}\;x=\frac{\varepsilon }{\varepsilon_{c}}\leq 1\\ \frac{x}{\alpha_{b}{\left(x-1\right)}^{2}+x}\;\;\;x=\frac{\varepsilon }{\varepsilon_{c}}\leq 1 \end{array}\right.$$

where $f_{c}$ is the compressive strength of concrete; $\varepsilon_{c}$ is the peak compressive strain, $\alpha_{a}$ rising section parameter, and $\alpha_{b}$ is the falling section parameter.

The basic parameters of the relevant mortars are shown in Table 6.

### 2.8. FTC Test

Different materials have different heat transfer rates, which can cause uneven temperature distribution inside the RC, resulting in differences in internal temperature stresses and crack expansion. The cohesive element is selected for the transition zone of the concrete model interface, the cohesive heat transfer effect is considered in the temperature transfer, and the temperature-displacement coupling solution is selected for the thermal sequence coupling solution of the model. Using ABAQUS for FTCs, the temperature solution mode needs to be set first, and this paper adopts a transient solution. After determining the single cycle time, the total FTC time is calculated for the setting. Subsequently, the boundary conditions are set, and the fixed method is a hinge support constraint at one end and a displacement constraint in the Y-axis direction at the other end; this constraint method cannot affect the temperature expansion and contraction of concrete. The temperature conditions are then set to apply a temperature load of 20 °C to the model boundary, and the load is set to cyclic using an amplitude table; i.e., the external temperature is applied at ±20 °C, with an initial temperature of 0 °C and an absolute zero of −273.15 °C. After determining the above settings and modifying the cell type, the COH2D4T heat transfer element was used to represent the ITZ. The calculated temperatures of each node were imported into the uniaxial compression experiment to obtain the compressive load capacity after the FTC.

In the temperature calculation of finite elements, the set external heat source continuously transfers heat to the model interior through the heat transfer medium. Different element material properties can cause inconsistencies in temperature transfer rates, as well as storage temperatures, resulting in differences in the linear expansion of the concrete model. During the FTC, the set mortar elements shrink when the temperature decreases. The ice property elements expand, and the expansion is converted into a stress field by ABAQUS calculations. When this temperature stress gradually increases and reaches the maximum fracture energy of the ITZ, the ITZ is damaged, and the stress is transferred to the mortar around the ice property element. After reaching the set plastic stage of the mortar element, the element begins to fail; i.e., ice expansion leads to concrete damage and cracking.

## 3. Results

The experimental data in this subsection were obtained from Wang [44], who studied the effect of rubber admixture in RC on its compressive strength and analyzed the mechanical properties of RC and ordinary concrete after FTCs. The literature shows that the relationship among the amount of rubber admixture, the rate of loss of compressive strength of concrete, and the rate of loss of compressive strength of FTCs can be obtained by conducting uniaxial compression and FTC experiments on the test specimens. This simulation uses this experimental material parameter to calculate the bearing capacity of the RC model under uniaxial compression and after FTCs and to compare the results with the experimental data. Suppose the difference between the numerical and experimental simulation results is within a reasonable range. In that case, it indicates that the model can provide a theoretical basis for the factors influencing the FTCs of RC to a certain extent.

The properties of the experimental cement are shown in Table 7. The coarse aggregates were selected from natural stones and continuously graded according to 5–20 mm. The experiment used equal volumes of 3- to 4-mm rubber particles to replace equal volumes of sand, with rubber substitution rates of 5%, 10%, and 15%.

Uniaxial compression experiments were conducted on plain concrete and RC, respectively, to compare the strength of RC with different admixtures and to obtain the relationship between the rubber replacement rate and compressive strength. Then, the fast freezing experiments were conducted on RC, and the strength of each experimental specimen was measured after 50, 100, and 150 cycles so that the addition of rubber to concrete and FTCs brought about by the damage could be modeled.

### 3.1. Uniaxial Compression Test and Simulation Result Comparison Verification

**Figure 8:**
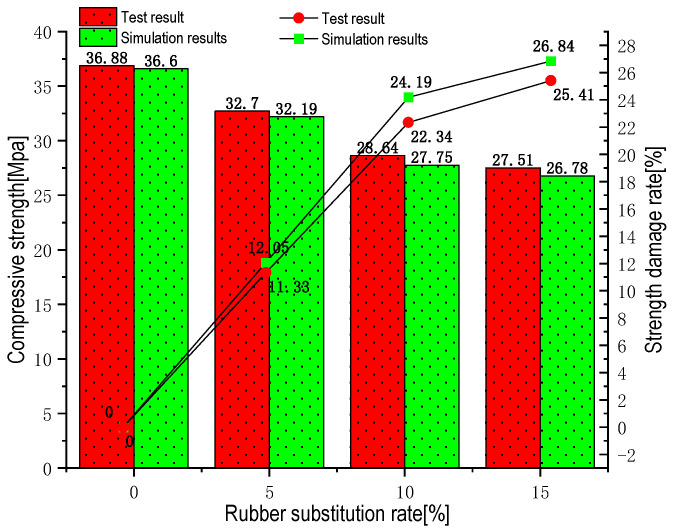
Comparative histogram of experimental data and numerical simulation of the compressive strength of rubber concrete under different substitution rates.

Based on the above simulation test method, the generated ordinary concrete model and RC model were calculated and analyzed to obtain the compressive strength under each admixture, which is shown in Figure 8. The figure shows that the concrete bearing capacity showed a decreasing trend with the increase in rubber aggregate admixture. At the beginning of the adding of the rubber, the concrete bearing capacity decreased rapidly, and then the decreasing trend slowed. When the rubber admixture was greater than 10%, the loss of compressive strength of concrete slowed, which is basically consistent with the experimental law of Wang scholars. Comparing the experimental data with the simulation results of this model, the maximum error rate of compressive strength of RC was 2.4%, and the maximum error rate of compressive strength loss was 1.7%, which is basically consistent with the experimental data and proves that the numerical simulation value of uniaxial compression of RC is more reliable.

### 3.2. Comparative Verification of Compressive Strength after FTCs

- 0% replacement rate RC

By analyzing the compressive strength of ordinary concrete before and after FTCs, the compressive strength of concrete did not decrease significantly after 50 FTCs, and it conformed to the bearing capacity strength of C35 concrete. When the number of FTCs increased to 100, the compressive strength decreased significantly. The trend of strength change in the simulation was basically consistent with the experimental results. The calculated compressive strength deviated from the experimental values, with a maximum deviation of 5.24%. The maximum deviation of strength damage value was 3.39%, but it was within the error range of numerical simulation calculations. Therefore, this model can effectively simulate the strength change process of ordinary concrete after FTCs.

- 5% replacement rate RC

After adding 5% rubber, the FTC damage of concrete was reduced compared to concrete without rubber, and the changing trend was consistent with the previous simulation. What is different is that the damage increased sharply after 50 cycles of ordinary concrete. In contrast, the strength damage percentage curve’s 5% replacement rate RC slope showed a significant upward trend after 100 FTCs. This phenomenon means that, after 100 FTCs, 5% of RCS suffered more severe freeze-thaw damage. Comparing the numerical simulation strength and experimental strength, the maximum deviation between the two was 2.4%, and the maximum deviation of strength damage was 2.7%, within the acceptable error range.

- 10% replacement rate RC

Concrete with 10% rubber further enhances the resistance of concrete to freeze-thaw damage, and the damage is more obvious after 100 FTCs. The simulation results are consistent with the experimental results, with a maximum compressive strength deviation of 4.19% and a strength damage deviation of 1.69%. The conclusion drawn is that the frost resistance of RC increases with increasing content when the rubber content does not exceed 10%.

- 15% replacement rate RC

**Figure 9:**
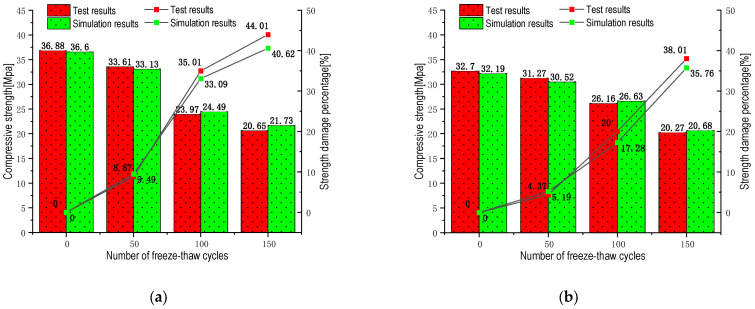

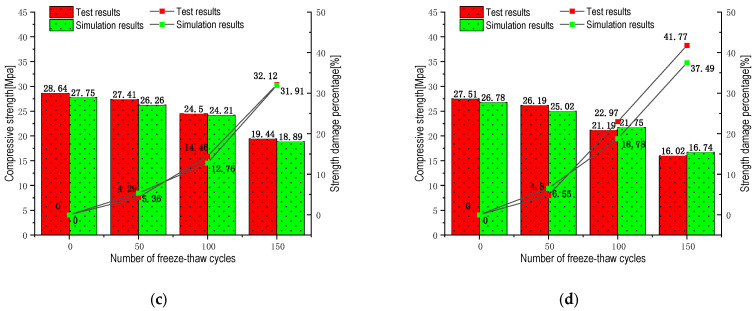
Comparative histogram of experimental data and numerical simulation of the compressive strength of rubber concrete under different substitution rates before and after FTC. (**a**) 0% rubber substitution rate; (**b**) 5% rubber substitution rate; (**c**) 10% rubber substitution rate; (**d**) 15% rubber substitution rate.

The slope of strength loss in the Figure 9 shows that the frost resistance of 15% RC is slightly weakened and no longer increases with the dosage increase. The maximum difference in compressive strength between the experimental and simulated results is 4.48%, and the deviation in strength loss is 4.27%, within an acceptable range. By calculating and comparing the experimental data results of the four models mentioned above, the model basically conforms to the numerical variation law of RC FTC.

By comparing the above experimental results with the numerical simulation results, the maximum error between them is 5.24%, which is within the acceptable range. The results show that the thermal model established in this study can better simulate the changes in the increases and decreases in the compressive strength of rubber concrete before and after FTZ, verifying the feasibility of the model. It also provides data support for the analysis, which can better study the subsequent uniaxial compressive crack expansion and temperature field distribution at the fine view level.

## 4. Discussion

### 4.1. RC Compressive Cloud Analysis

**Figure 10:**
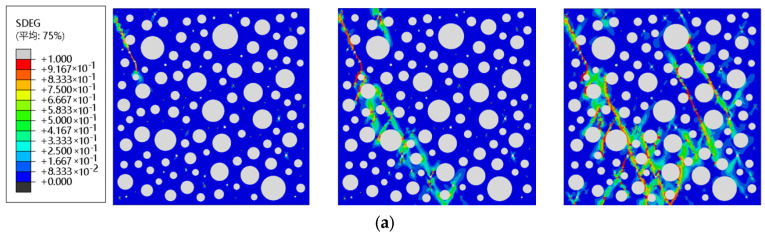

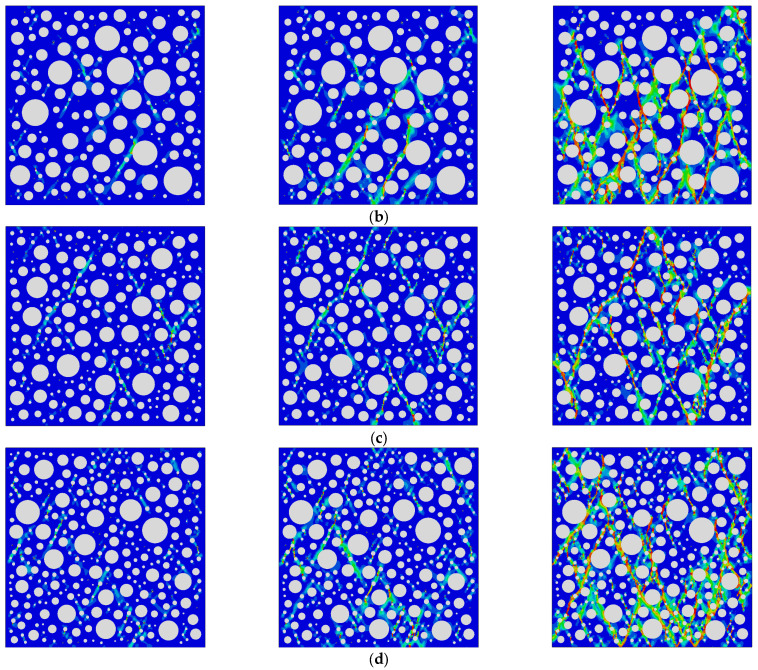
Uniaxial compression damage clouds for each substitution rate of rubber: (**a**) 0% rubber substitution rate; (**b**) 5% rubber substitution rate; (**c**) 10% rubber substitution rate; (**d**) 15% rubber substitution rate. From left to right: at two-thirds of the maximum strain, at the maximum strain, and at the residual strain. (平均: Average).

The ABAQUS damage cloud diagram uses the SEDG indicator to determine the degree of RC damage, and the value of SEDG ranges from 0 to 1. When it reaches 1, it indicates that the element is completely damaged there. The compressive damage cloud for each mix of rubber concrete is shown in Figure 10.

During the loading process of the 5% low substitution rate RC model, the water-mortar ITZ was the first to be damaged, followed by the mortar-rubber ITZ, which was damaged and extended to the surrounding mortar. The reason for this outcome is that the Poisson’s ratio of the rubber material is much larger than that of the mortar. The Poisson effect generated by the compression deformation of the rubber is directly transferred to the weak mortar-rubber ITZ, causing the small cracks in the RC to start here. The stress concentration generated destroys the mortar around the rubber particles. As the compressive load increases, the cracks unfold in the oblique vertical direction, and the mortar-aggregate ITZ also begins to break down, mainly in the direction of the line between the center of the rubber particles and the surrounding near-aggregate. The cracks located between the aggregate and the rubber extend into the damaged aggregate and rubber ITZ, as can be seen from the stress cloud diagram, showing that the cracks mainly occur near the dense area of large diameter aggregate and rubber. Low substitution rate rubber distribution is more dispersed, with cracks initially unfolding in part of the rubber above and below, and a small amount of rubber above and below both produce cracks, while very little rubber near did not appear cracks. When the loading reached two-thirds of the maximum strain, the rubber at the beginning of the cracks was produced by the extension of the oblique vertical direction, and the presence of a relatively small number of rubber particles only occurred in the ITZ damage. When the maximum strain was reached during loading, cracks extending toward nearby rubber particles occurred at the ITZ of the mortar aggregate. More initial cracks at the rubber appeared with the initial cracks of another section of rubber to produce joint enlargement cracks. Some cracks in the mortar appeared to extend along the tangential direction of the rubber-aggregate ITZ unfolding. When the residual strain was reached, the RC was completely crushed, and the crack damage form showed minor conical damage according to the typical concrete damage type under compression. Still, the incorporation of rubber led to no obvious major cracks, which guarantees the integrity of the RC after being compressed.

Compared with the 5% substitution rate, the 10% substitution rate resulted in reduced internal spacing and good distribution, and the stressed regions showed more substantial integrity. When loaded to two-thirds of the maximum strain, the damage of the model was similar to that of the 5% substitution rate, with the initiating cracks appearing at both ends of the rubber and multiple mortar-rubber ITZs produced by cracks connected in the direction of the line connecting the center of the damaged rubber transition zone with the center of the nearby damaged rubber ITZ. Compared to the 5% replacement rate rubber, the 10% damage occurred much later, with significantly more unconnected microscopic cracks, rapidly expanding cracks at maximum strain, a wider distribution of cracks and extension into the dense rubber zone, and a significantly greater number than with 5% RC. The residual strain was reached, no large penetration cracks were evident, microscopic cracking increased, and resistance to damage improved, indicating that adding the rubber material resulted in the RC having better integrity after compressive stress.

The initial crack unfolding of 15% high-substitution RC was similar to that of low-substitution RC, with cracks appearing in the mortar-rubber ITZ and the mortar region near the rubber in sequence, followed by the extension of the mortar-aggregate ITZ to the mortar region around the aggregate. When loading reached two-thirds of the maximum strain, more joint cracks and dispersed cracks formed in the internal areas of the concrete than in the RC with low replacement rates. When reaching the maximum strain, most of the rubber had a linkage effect with the surrounding rubber, the mortar-aggregate ITZ was destroyed, more long cracks were produced in the internal mortar region, and the crack direction was the same as the tangent direction of the already destroyed rubber and aggregate ITZ. After reaching the final damage, the number and width of cracks were significantly greater than those of the low substitution rate rubber, and the integrity was well maintained, with more cracks distributed around the large grain size aggregate and more uniform distribution of conical cracks at the bottom.

### 4.2. The Effect of Temperature Transfer on RC

**Figure 11:**
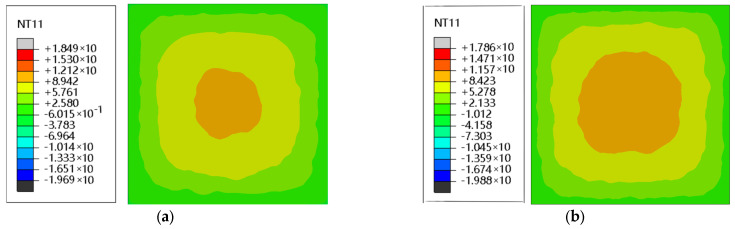
Temperature field distribution of 0% substitution and 10% substitution RC at the same moment: (**a**) 0% rubber substitution rate; (**b**) 10% rubber substitution rate. TN11 is the node temperature.

To analyze the distribution of the internal temperature field of RC, the RC with 0% substitution and 10% substitution were taken for comparison and exploration. Figure 11 shows the temperature field distribution of the two substitution rates simultaneously: the 0% substitution and 10% substitution boundary temperatures are −0.0522 °C and −0.0479 °C respectively, which means that the concrete is being loaded at a low temperature; the central temperature of ordinary concrete is 10.129 °C, and the difference between the inner and outer temperature is 10.18 °C. After adding 10% rubber admixture, the central temperature drops to 9.725 °C, and the difference between the inner and outer temperatures is smaller than that of ordinary concrete, which is 9.77 °C, indicating that ordinary concrete is more affected by temperature effects. Selecting the aggregate center node temperature, we calculate the two admixtures from the center of the maximum temperature down to the minimum temperature per degree of decline in the length of time required (Table 8). The center of the concrete containing rubber had positive and negative temperatures lower than those of ordinary concrete, and the heat transfer time was greater than that of ordinary concrete. In large projects, as the volume of concrete increases, the temperature difference between inside and outside will gradually increase, the heat transfer time also gradually increases, and the effect of temperature on RC also increases, which has not been noted by many scholars. The reason for this outcome is that the heat transfer rates of rubber materials and mortar materials differ greatly. The heat transfer rate of rubber is only one-fifth that of mortar materials, and the specific heat capacity of rubber materials is much greater than that of mortar, causing RC in the external temperature to change, better hindering the temperature transfer, reducing the temperature change caused by linear expansion, and thus reducing the stress increase; thus, RC will be able to play a better role in the FTC of external conditions.

### 4.3. Uniaxial Compression Damage Clouds after FTCs

**Figure 12:**
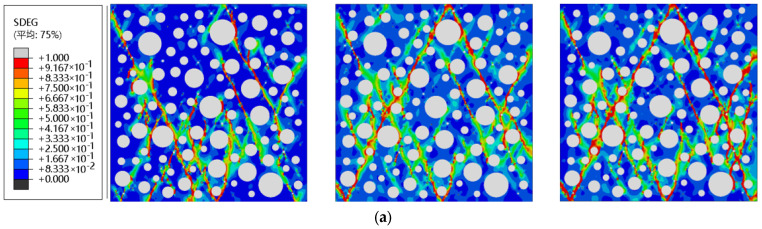

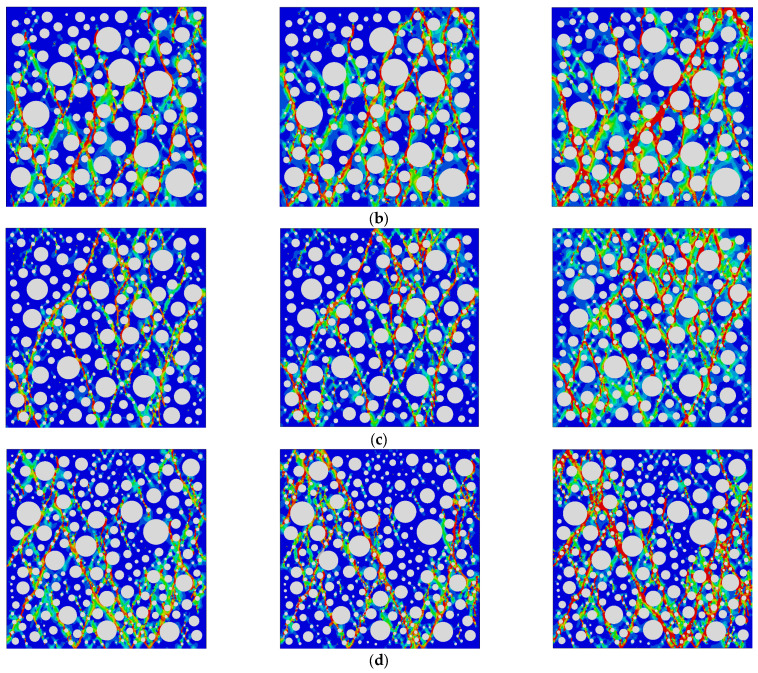
Uniaxial compression damage clouds after FTCs for each dose of RC: (**a**) 0% rubber substitution rate; (**b**) 5% rubber substitution rate; (**c**) 10% rubber substitution rate; (**d**) 15% rubber substitution rate. from left to right: 50 FTCs, 100 FTCs, 150 FTCs. (平均: Average).

Each admixture’s RC after different FTCs was subjected to uniaxial compression, and the final damage clouds are shown in Figure 12. By comparison, the crack width and length of RC increased with the number of FTCs.

After 50 FTCs of RC with a 0% substitution rate, the crack on the left side occurred and extended toward the top center; after 100 cycles, the concrete material was severely damaged, the lower crack penetrated and joined with the middle and upper cracks, and the specimen formed two major conical damage cracks; after 150 cycles, the damage did not change much, and the crack width increased. After 50 FTCs of RC with a 5% substitution rate, a crack appeared on the right side. After 150 cycles, the material damage was more serious, and the main crack was obvious after pressure, which means that the FTC of RC with 5% substitution rate was greater than 100 times, the frost resistance was weakened, and the damage was greater. After 50 cycles, some of the cracks widened, causing a small number of crack extensions; with 100 FTCs, the upper right side of the crack extended more, creating more tiny cracks; when the FTC reached 150 times, the upper part also showed the formation of many scattered cracks and downward extension, but still for the formation of the main crack, it was maintained better overall. In RC with a 15% substitution rate after 50 FTCs, some cracks continued to extend, and a small amount of crack damage was aggravated; after 100 cycles, the damage was significantly aggravated due to more micro-crack extension, resulting in the formation of the main crack during compression; after 150 cycles, there were more 100 times more undamaged cracks, with a large number of cracks obviously extending, consistent with the results for compressive strength in Figure 9.

In the uniaxial compressive damage cloud, shown in Figure 10 and Figure 12, there is an uneven distribution of some cracks. The reason is that the randomly generated aggregate can restore the non-homogeneous structure inside the concrete to a certain extent, but it leads to some fine water or rubber being more concentrated. Due to the weak compressive properties of fine water and rubber, the concentrated area belongs to the weak compressive zone of the overall RC model. When subjected to external loading, cracks develop rapidly toward the concentrated area, resulting in significantly more cracks on one side than the other, especially after FTCs are performed. However, the damage pattern is also more in line with the real situation, with cracks being denser and the damage being more severe near the rubber, void water, and large aggregates.

### 4.4. Stress–Strain Relationship of RC before and after FTCs

**Figure 13:**
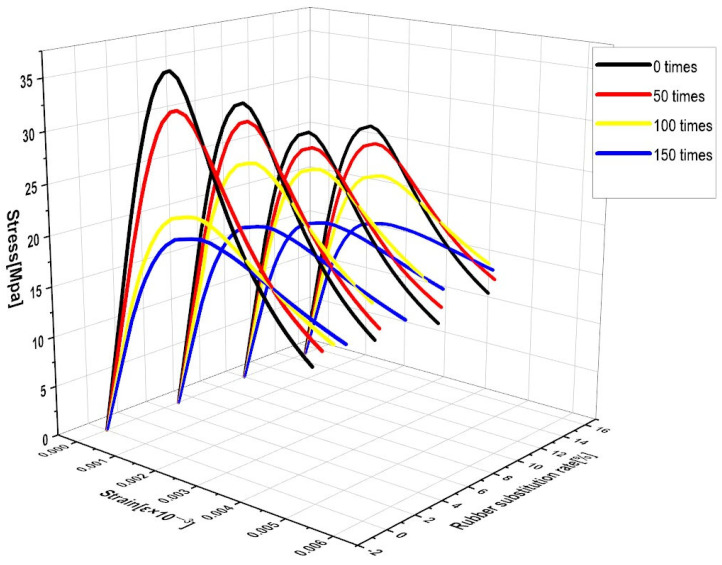
Uniaxial compressive stress–strain curves of RC before and after FTCs for each dose.

The uniaxial compressive stress–strain curves (Figure 13) were analyzed before and after the model FTCs, and the processes were mainly the elastic, plastic, and damage phases. As the number of FTCs for each dose of RC increased, the slope of the elastic phase also decreased, implying that the FTCs continuously decrease the material elastic modulus. For the 0% substitution rate of RC, it is obvious that the stress decreased significantly in 50–100 FTCs, while the stress decreased in 5%, 10%, and 15% mainly in 100–150 FTCs. The strain increased with the number of FTCs, and the strain growth values were 0.00015/0.00054/0.00065 for 0% substitution rate RC FTCs 50/100/150, 0.000105/0.000301/0.000523 for 5% substitution rate RC FTCs 50/100/150 The strain growth values are 0.000085/0.000279/0.000432 for the 50/100/150 FTCs, 0.000111/0.000313/0.000537 for the 15% FTCs, and 0.000085/0.000279/0.000432 for the 10% FTCs, and 0.000111/0.000313/0.000537 for the 15% FTCs. About 10% of rubber is subjected to FTCs with smaller strain increases and is more resistant to FTC damage.

### 4.5. Concrete Freeze-Thaw Damage Prediction

The random distribution of materials inside the concrete model makes the formation of microscopic cracks stochastic in nature, coupled with the complexity of external temperature variations, which requires a special mathematical and statistical approach to solve the functional relationship between freeze–thaw damage and rubber admixture. The three-parameter Weibull distribution is widely used in predicting the probability of failure, and according to the three-parameter Weibull probability model, the expression of the density function for the service life of concrete in the FTC state is obtained as follows:

(15) $$f\left(\theta \right)=\frac{\gamma }{\theta }\varepsilon^{\gamma -1}exp\left[-{\left(\frac{\varepsilon -\theta }{\theta }\right)}^{\gamma }\right]$$

Then its distribution function is:

(16) $$F\left(\theta \right)=\int_{0}^{\varepsilon }f(\varepsilon )d\varepsilon =1-exp\left[-{\left(\frac{\varepsilon -\theta }{\theta }\right)}^{\gamma }\right]$$

where γ, θ, ε are Weibull shape parameters.

To predict the residual strength of RC after temperature damage, this article establishes the relationship between strength loss value and FTCs to determine the fatigue degree of RC. A least-squares fit was performed using the Weibull probability model. After considering the damage threshold, the empirical formula is a nonlinear quadratic correlation between the strength loss value and the number of FTCs, so it is assumed that it fits the following formula [45]:

(17) $$\vartheta =AN^{2}+BN+C$$

where ϑ is the strength loss value; A, B, and C are constants; and N is the number of FTCs.

**Figure 14:**
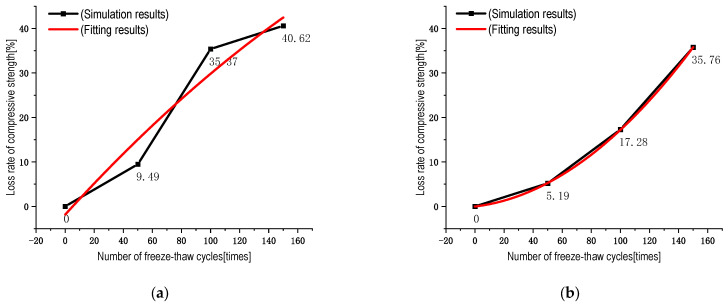

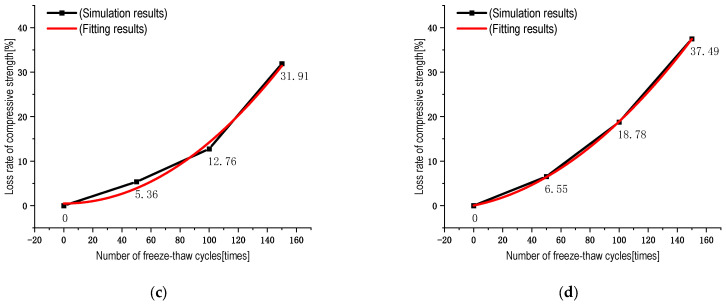
The fitting curve of strength loss of RC in FTCs: (a) 0% rubber substitution rate; (b) 5% rubber substitution rate; (c) 10% rubber substitution rate; (d) 15% rubber substitution rate.

The fitted equations are shown in Table 9. The fitted graph is shown in Figure 14, and the fitted curve R^2^ meets the requirements. It is found that the fitted curve is closer to the trend the FTC strength loss of RC, which has some reference significance for the subsequent load-bearing strength damage of RC.


$$\theta_{0}=-4.2447e^{-4}N^{2}+0.35915N-1.85075$$

$$\theta_{5}=0.00133N^{2}+0.03944N-0.02594$$

$$\theta_{10}=0.00138N^{2}-5.4217e^{-4}N+0.48464$$

$$\theta_{15}=0.00122N^{2}+0.067N+0.04$$

### 4.6. Potential Applications and Developments

This study establishes a thermally coupled RC model, which contains rubber, mortar, pore water, and aggregate multiphase materials inside it, which is a more novel numerical simulation model. The study analyzed the effect of temperature on the compressive strength of RC. It verified the reducibility and feasibility of the model by analyzing the strength loss and damage cloud diagram of uniaxial compression. The model can also be used to analyze the thermodynamics of concrete with other admixture materials by changing the internal material composition, the size of admixture materials, and the thermodynamic properties, providing diversified ideas for related studies.

In this study, voids are defined as pore water for thermal analysis. Still, it is found that the introduction of cohesive elements as ITZs and the generation of a small number of voids in uniaxial compression simulations will reflect the actual damage form better than the traditional uniaxial compression model, rendering the simulated damage process closer to the test and improving the accuracy of the simulation.

In this study, a two-dimensional calculation model was used. With the improvement of computer performance and analytical capability, the research and development of a three-dimensional calculation model can better study the freeze-thaw damage of RC and achieve a higher level of accuracy.

## 5. Conclusions

A thermodynamic calculation model of RC was established using the ABAQUS analysis platform. The effect of the rubber admixture on frost resistance was analyzed by the changes in the increases and decreases in uniaxial compressive strength before and after FTC, and the accuracy of the model was verified by comparing the calculated results of the compressive strength of concrete before and after FTCs with the experimental results. The results of this study are as follows.

The RC model established in this paper adopts a relatively novel research method in uniaxial compression and temperature transfer and effectively compensates for the defects of the traditional uniaxial compression model through the use of pore and cohesive elements, providing a reference basis for the numerical simulation of other types of polymer concrete. Adding rubber to concrete can impede temperature transfer and effectively improve the damage caused by FTCs. After FTCs of RC with 0%, 5%, 10%, and 15% admixtures, the strength loss rates are 40.62%, 35.76%, 31.91%, and 37.49%, respectively, and the rubber admixture at 10% has a better ability to resist damage by FTCs. The addition of rubber to concrete causes the original compressive strength of concrete to decrease, and the larger the rubber admixture, the lower the compressive strength. RC is subjected to external compressive loads to produce more micro-cracks than ordinary concrete, mainly concentrated in large aggregate and rubber-dense areas. However, adding rubber will destroy the concrete’s integrity and is less likely to form main cracks.

## Figures and Tables

**Table 1:** Total area generated by different diameter aggregates in the model.

Rubber Replacement Rate [%]	5–10 mm [mm^2^]	10–15 mm [mm^2^]	15–20 mm [mm^2^]	Rubber [mm^2^]
0%	2933	2069	1217	0
5%	2933	2069	1217	364
10%	2933	2069	1217	728
15%	2933	2069	1217	1092

**Table 2:** Thermodynamic parameters of RC.

Parameter	Element	Mortar	Aggregate	ITZ	Rubber	Pore Water
Modulus of elasticity	GPa	30	43	-	0.7	0.88
Poisson’s ratio		0.2	0.16	-	0.49	0.33
Density ^1^	kg/m^3^	2.4 × 10^3^	2.7 × 10^3^	-	1.1 × 10^3^	0.98 × 10^3^
Specific heat capacity ^1^	J/(g K)	1.05	0.97	0.58	1.7	2.1
Thermal conductivity ^1^	W/(m-k)	0.93	3.5	0.73	0.2	2.16
Thermal expansion ^1^	1/°C	1 × 10^−5^	0.7 × 10^−5^	1.2 × 10^−5^	1.15 × 10^−4^	2 × 10^−4^

^1^ It comes from the literature [28,29,30,31].

**Table 3:** The number of cell nodes and rate of change in the strength of RC with 0% substitution rate at different mesh sizes.

Mesh Size	Number of Nodes	Number of Elements	Compressive Strength	Rate of Change
1.25 mm	22,722	42,497	35.53	-
1.5 mm	16,384	30,317	35.83	0.8%
1.75 mm	12,470	22,870	35.96	1.2%
2 mm	10,083	18,340	39.03	9.8%
2.5 mm	7545	13,542	42.68	20.12%

**Table 4:** The number of cell nodes and rate of change in the strength of RC with 10% substitution rate at different mesh sizes.

Mesh Size	Number of Nodes	Number of Elements	Compressive Strength	Rate of Change
1.25 mm	27,009	49,749	27.21	-
1.5 mm	21,484	39,151	27.46	0.9%
1.75 mm	17,496	31,593	27.75	1.9%
2 mm	14,467	25,902	35.46	30%
2.5 mm	10,592	18,690	40.92	50.39%

**Table 5:** Cohesive parameters of the model.

	Normal Strength (MPa)	Shear Strength (MPa)	Normal Fracture Energy (N/mm)	Shear Fracture Energy (N/mm)
Aggregate-Mortar ITZ	2.6	10	0.025	0.0625
Rubber-Mortar ITZ	1.82	7	0.0175	0.0438
Pore water-mortar ITZ	0.26	1	0.0025	0.00625

The aggregate-mortar ITZ reference [42], rubber-mortar ITZ, and void water-mortar ITZ for the results are obtained after several trial calculations.

**Table 6:** Plastic damage parameters of mortar materials.

Expansion Angle ψ	Eccentricity	$\mathrm{Stress}\;\mathrm{Ratio}\;f_{bo}$ $/f_{co}$	K
30	0.1	1.16	0.6667

**Table 7:** Table of relevant physical properties of experimental cement.

Standard Consistency %	Setting Time Min		Flexural Strength		Compressive Strength	
24.2	Initial setting	Final condensation	3 days	28 days	3 days	28 days
	180	270	5.8	9.2	22.5	48.2

**Table 8:** Maximum and minimum temperatures and cooling times with 0% substitution rate and 10% substitution rate RC centers.

Rubber Admixture [%]	Center Maximum Temperature [°C]	Center Minimum Temperature [°C]	Duration of Temperature Drop [s]	Duration of Drop per Degree Celsius [s]
0%	10.626	−10.618	3479	163.76
10%	10.444	−10.544	3609	171.95

**Table 9:** Fitting function of strength loss of RC in FTCs.

Rubber Replacement Rate	Fitting Function	R^2^
0%	$-4.2447e^{-4}N^{2}+0.35915N-1.85075$	0.94
5%	$0.00133N^{2}+0.03944N-0.02594$	0.999
10%	$0.00138N^{2}-5.4217e^{-4}N+0.48464$	0.991
15%	$0.00122N^{2}+0.067N+0.04$	0.999

## Data Availability

The data used in the article can be obtained from the author here.
